# Temperatures around conception affect metabolic health in adulthood

**DOI:** 10.1038/s43856-026-01496-8

**Published:** 2026-03-27

**Authors:** Timo S. Münz, Fabienne Pradella, Nathalie J. Lambrecht, Sabine Gabrysch, Reyn van Ewijk

**Affiliations:** 1 Chair of Statistics and Econometrics, Johannes Gutenberg University Mainz, Mainz, Germany; 2 Heidelberg Institute of Global Health, Heidelberg University, Heidelberg, Germany; 3 Division of Primary Care and Population Health, Department of Medicine, Stanford University, Stanford, CA USA; 4 Institute of Public Health, Charité—Universitätsmedizin Berlin, Corporate Member of Freie Universität Berlin and Humboldt-Universität zu Berlin, Berlin, Germany; 5 Research Department 2, Potsdam Institute for Climate Impact Research (PIK), Member of the Leibniz Association, Potsdam, Germany; 6 Center for Innovation in Global Health, Stanford University, Stanford, CA USA

**Keywords:** Epidemiology, Metabolic disorders

## Abstract

**Background:**

Epigenetic adaptations around conception can help organisms adjust to their future environment. Pre-conception cold exposure is thought to increase active brown fat mass, and as brown fat metabolizes stored energy to generate heat, this helps adjust to life in cold environments. We examine the implications of this process for human metabolic health.

**Methods:**

We use data on 437,504 individuals born between 1934 and 1971 from the UK Biobank, and match these to historical temperature data. To isolate causal impacts of temperature, we utilize day-specific temperature deviations during the calculated pre-conception period relative to the long-term mean temperature for the same location and day of year. This approach leverages a quasi-random variation in temperature. Associations between pre-conception temperature exposure and adult metabolic health were estimated using regression models adjusted for relevant covariates.

**Results:**

Individuals conceived when temperatures were lower than usual have lower body mass indices, smaller waist circumferences, and lower levels of triglycerides and total cholesterol in adulthood, while the effect on glycated hemoglobin appeared to be less strong.

**Conclusions:**

Our study indicates that pre-conception environmental conditions can influence human metabolic health, potentially through epigenetic mechanisms linked to brown fat activity. These findings have implications for potential health effects of climate change, and, more strongly, of improved indoor insulation.

## Plain Language Summary

We combined UK Biobank data from over 430,000 people with historical weather records, examining whether unusually cold or warm conditions before conception have lasting health effects. Previous research suggests that when fathers experience cold conditions before conception, their offspring may have more active brown fat, a type of fat that produces heat.

Our study shows that the temperature around the time a person is conceived influences their metabolic health later in life. People conceived during colder-than-usual periods tend to have lower body weight, smaller waists, and healthier levels of blood fats (triglycerides and cholesterol) as adults. For example, in an average adult, a 2 °C lower temperature at conception is associated with a 126 g lower body weight.

As global temperatures rise and better housing insulation keeps homes warmer during winter, long-term consequences for metabolic health can be expected.

## Abstract (web summary)

Münz et al. link historical temperature data to over 437,000 UK Biobank participants to test how pre-conception cold exposure affects later metabolic health. Individuals conceived during colder-than-usual periods show lower adult BMI, waist circumference, and blood lipid levels, suggesting lasting epigenetic effects.

## Introduction

Humans with similar sets of genes live in diverse climates that change over time. Epigenetic mechanisms provide a relatively fast way of biological adaptation to the environment^1^. While such processes occur throughout life, they tend to be most pronounced during early development, making environmental conditions during this stage potentially relevant for later health^2^. Most previous research has focused on adverse prenatal exposures such as malnutrition^3–5^, stress^6^, and pollution^7^, leading to worse health outcomes in later life, often persisting decades after the initial exposure^8–12^. But epigenetic processes in response to environmental cues can also be beneficial and act as adaptive strategies that prepare the organism for anticipated similar future environments^9,10^.

This paper investigates the implications of ambient temperatures before conception on metabolic health in adulthood. Paternal exposure to colder temperatures before conception is thought to set in motion an epigenetic mechanism during sperm production that stimulates the formation of brown fat, or brown adipose tissue (BAT), in the offspring^13^. This can serve as an adaptation mechanism that prepares the organism for life in a colder environment^9,10^. Unlike white fat, which stores energy in the form of lipids, BAT is characterized by its ability to actively metabolize stored energy to generate heat through the process of non-shivering thermogenesis. Activation of BAT is primarily triggered by colder temperatures and involves converting chemical energy from fatty acids and glucose into heat, serving as a defense against cold^14–19^. BAT is thus integral to the complex regulation of body temperature in mammals, including humans.

A positive side effect of these properties of BAT is that individuals with higher BAT mass and activity tend to have lower BMI and blood glucose concentrations^17,18,20–23^. Additionally, through the burning of fatty acids, BAT activation results in lower levels of plasma triglycerides and cholesterol^14,19,24–27^. This underscores the recognized potential of BAT as a therapeutic target for obesity and related metabolic diseases like type 2 diabetes^14,17–19,28–35^.

While there is very little research linking cold exposure to later-life metabolic outcomes, Sun et al.^13^ show that mice whose parents were exposed to 8 °C before mating exhibited increased BAT activity, resulting in higher metabolism and lower obesity, even on a high-calorie diet, compared to a control group kept at 23 °C. Notably, this effect is observed with paternal, but not maternal exposure, suggesting epigenetic changes through sperm cells. Sperm from cold-exposed males had reduced methylation at the Adrb3 gene, which encodes the ${\beta }_{3}$-adrenergic receptor crucial for BAT activation. This reduced methylation likely increases Adrb3 expression in offspring, boosting their BAT activity and ability to generate heat. Further supporting their findings, Sun et al.^13^ analyzed PET/CT scans of about 8500 Swiss individuals, finding a higher likelihood of active BAT in adulthood among those conceived in colder months of the year, typically correlating with lower BMI.

However, evidence on whether cold exposure before conception affects human metabolic health outcomes other than BMI is still lacking. Moreover, using the month of conception as an indicator of cold exposure presents challenges in isolating the effect of temperature. The season of conception is correlated with sociodemographic characteristics, which are again predictors of metabolic health^36^. This correlation is partly due to seasonality in the work calendars of employed mothers^37^ and partly due to biological processes. Specifically, human biological functions respond to seasonal variation in daylight length, although the degree of responsiveness differs between individuals^38,39^. While sexual activity levels are relatively constant throughout the year—except for peaks around major cultural or religious celebrations—female fertility fluctuates with the seasons. Environmental light intensity is thought to play an important role in this variation^40,41^.

Research on the relationship between pre-conception cold exposure and long-term metabolic health in humans relies on observational data and therefore faces challenges in establishing causal evidence. Two straightforward solutions would be comparing people born in locations with different climates or people conceived during different seasons. However, the former solution is problematic since the place of birth is correlated with many other factors. The latter solution is also problematic because the season of conception correlates with a broad array of measurable and unmeasurable relevant parental characteristics. To overcome these challenges, we utilize the variation in temperatures for specific places on specific days of the year. For example, January 15th in a specific location will be comparatively colder in some years and warmer in others. We use this quasi-random variation over the years, comparing individuals conceived at the same time of the year in the same area.

We use data from the UK Biobank on the metabolic health of about 500,000 adults. The study participants were born between 1934 and 1971, and their assessments at the survey center took place from 2006 to 2010^42^. For our analysis, we focus on a subset of about 437,500 participants who provided mappable birthplace information and are non-adopted. Details of the sample selection are provided in the “Methods” section. The metrics evaluated include body mass index (BMI, kg/m^2^), waist circumference (WC, cm), glycated hemoglobin (HbA1c, mmol/mol) as a marker of the average blood sugar level of the past 2–3 months, triglycerides (mmol/L), and total cholesterol (mmol/L), all key indicators of metabolic health^43–49^. We combine these health data with historical temperature data from the Met Office Integrated Data Archive System (MIDAS)^50,51^, employing the geographical coordinates of the study participants’ birth locations to capture temperature conditions relevant to conception.

During the final 1–2 weeks of spermatogenesis, sperm cells acquire small RNAs while passing through the epididymis, a process that serves as epigenetic priming prior to fertilization^52,53^. Consequently, this period immediately before conception is crucial, as environmental factors affect the sperm cells’ epigenome, leading to epigenetic alterations in the physiology of future offspring. However, since mature sperm can be stored in the male reproductive tract for some time and can also survive several days within the female genital tract before fertilization occurs, the effective time window of environmental sensitivity may extend beyond these 2 weeks^54,55^. Therefore, we define the temperature during the 2 weeks leading up to conception as a critical timeframe for our research, while acknowledging that relevant exposures could occur somewhat earlier as well.

Given the natural variation in pregnancy length and given preterm births, the exact time of conception cannot be precisely determined. We thus estimate the date of conception (DOC) by deducting 266 days, the average length of gestation^56^, from the birth date of UK Biobank study participants. Additionally, there is uncertainty not only about the exact time of conception but also about the specific biological period during which epigenetic priming occurs. Previous research furthermore gives no indications about the duration of temperature exposure that is needed to exert an effect. While previous evidence suggests that the 2 weeks before conception are relevant, this does not exclude the possibility that epigenetic priming may occur at other stages of spermatogenesis. To address these uncertainties and capture relevant exposure periods, we calculate average temperature deviations for five different timeframes: at the estimated DOC, 2 weeks before conception till estimated DOC (−2 w; DOC), from 3 weeks before to 1 week after conception (−3 w; +1 w), from 4 weeks before to two weeks after conception (−4 w; +2 w), from 5 weeks before to 3 weeks after conception (−5 w; +3 w). Each exposure measure comes with its own set of advantages and disadvantages. Shorter intervals can provide more precise estimates by minimizing noise, but they may risk missing relevant exposure periods due to variability in gestational length and uncertainties in biological timing. Conversely, longer intervals are more likely to capture the relevant biological period but introduce additional noise, potentially diluting effect sizes by including irrelevant exposure periods. The longest time periods include one or more weeks after the estimated DOC. It seems biologically plausible that ambient temperatures before and during conception play a more important role than after conception, as paternal but not maternal cold exposure seems to matter^13^. However, the reason for including longer pre-conception periods lies in the higher likelihood of capturing the relevant period in situations where there is uncertainty in the estimated DOC, for instance, due to a preterm birth. By employing both narrow and broad intervals, we aim to balance the need for precise measurement with the necessity of capturing the relevant exposure period.

Our analysis consists of three steps. First, for each weather station and for each date between 1933 (estimated conception year of the oldest individual) and 1971, we determine the deviation from the long-term average temperature for that location and day of the year. It should be noted that this time frame coincided with a temperature-wise relatively stable climatic period in the United Kingdom (UK) that preceded a major upswing in temperatures starting in the 1980s^57^. Second, for each respondent, we calculate the mean temperature deviation for each of the pre-conceptual periods defined above. This involves using the data obtained in step 1 from all weather stations located within a 200-km radius of the respondents’ birthplaces, which were measured at a 1-km resolution. We apply an inverse distance weighting method to give more weight to weather stations closer to the respondents’ locations of birth, ensuring a more accurate representation of temperature exposure. As a robustness check, we also apply a nearest-neighbor approach, assigning temperature recordings from the closest weather station to each respondent’s birth location. In the third step, we regress the later-life metabolic outcomes on the temperature deviation before conception, controlling for area-by-month of birth, sex, year of birth, and year of assessment, for 172 administrative areas.

In our study, we find that people conceived during colder-than-usual periods show healthier metabolic profiles in adulthood. Lower pre-conception temperatures are associated with reduced body mass index, smaller waist circumference, and lower levels of glycated hemoglobin, triglycerides, and total cholesterol. Except for glycated hemoglobin, these effects are stronger when temperature exposure is measured over a longer periconceptual window. Effects remain after correction for multiple testing. The results are robust across several alternative models, sex-specific analyses, and tests for selective fertility. Overall, our findings suggest that colder environmental conditions around conception have lasting, beneficial effects on metabolic health. This likely reflects epigenetic adaptations that enhance brown fat activity and energy regulation. The study underscores how environmental conditions at conception can shape long-term health, with important implications in the context of climate change and modern living environments.

## Methods

### Individual-level health data from the UK Biobank

**Fig. 1:**
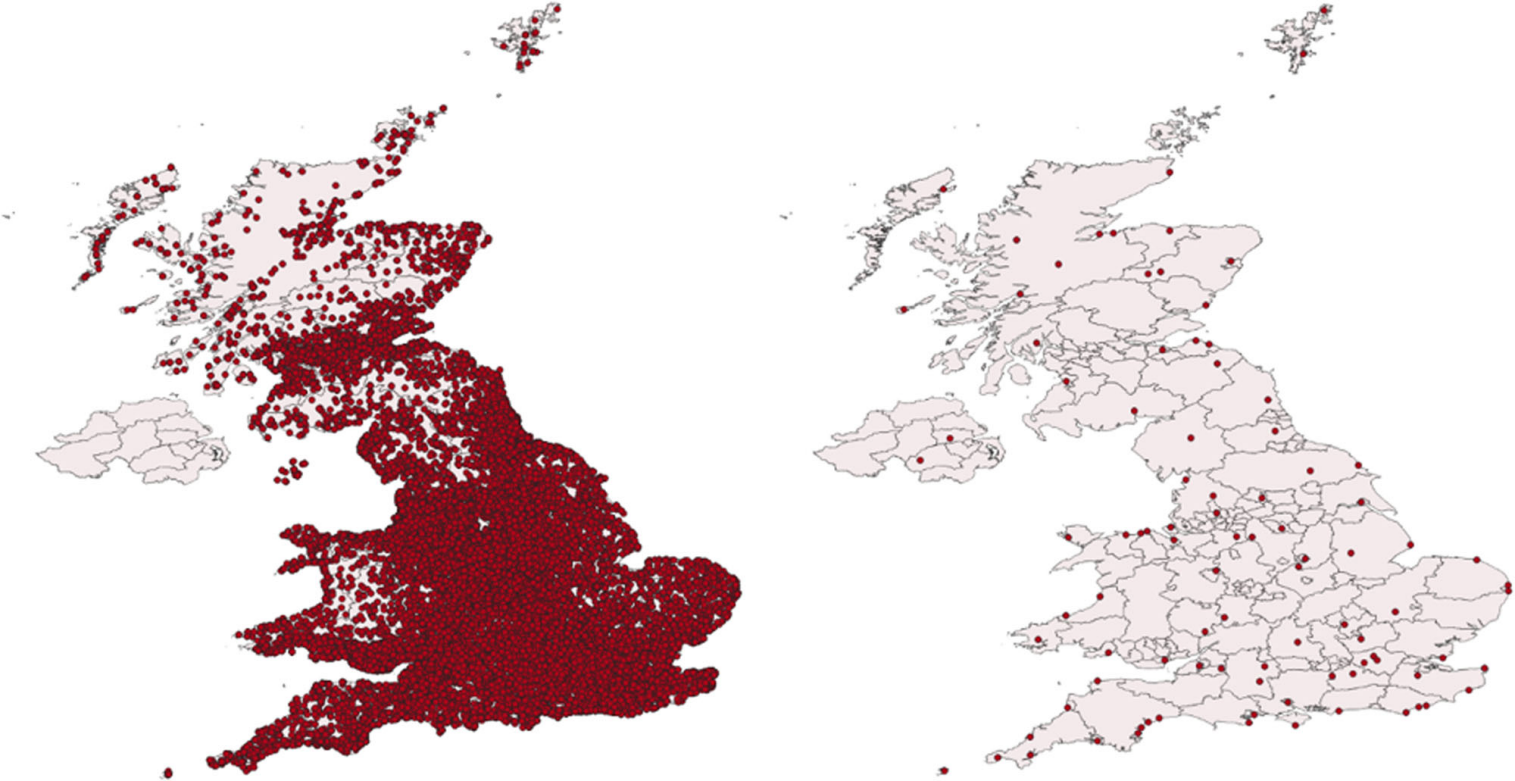
Geographic distributions of birthplaces and weather stations. Birth locations of 437,504 UK Biobank participants (left panel, red dots) and the positions of 94 MIDAS Open weather stations (right panel, red dots), each with at least 30 years of temperature data between 1933 and 1971. Maps are created using QGIS version 3.16.14-Hannover. Administrative boundary polygons correspond to 2^nd^-level administrative areas, derived from GADM version 3.6 shapefiles (https://gadm.org/index.html).

Our research utilizes detailed health data sourced from the UK Biobank, an extensive biomedical database. This dataset includes records from approximately 437,500 adults, assessed between 2006 and 2010, who were born between the years 1934 and 1971 in the United Kingdom and resided in the United Kingdom at the time of assessment. The following elements were part of the assessment center visit process: touch screen questionnaires, a face-to-face interview, anthropometric measurements such as BMI and waist circumference, as well as sample collection of blood, urine, and saliva. The UK Biobank provides participants’ birth dates and locations in Easting and Northing coordinates (Fig. 1). The UK Biobank records the birthplace of individuals through a question posed to the participants born in England, Scotland, or Wales. Participants were asked: “What is the town or district you first lived in when you were born?” We use this location as a proxy for the place of conception, assuming that for most participants, conception occurred nearby. Based on the participant’s response, the interviewer selected the corresponding location from an extensive and detailed list of around 43,000 place names in the UK. This birthplace information was then converted into Easting and Northing coordinates at a 1-km resolution.

### Metabolic health outcomes

Our analysis focuses on five indicators related to metabolic health: the anthropometric measures BMI (*kg/m²*) and waist circumference (*cm*), as well as blood levels of glycated hemoglobin (HbA1c, *mmol/mol*), triglycerides (*mmol/l*), and cholesterol (*mmol/l*).

### Study sample

The initial sample covers 452,149 participants from England, Scotland, and Wales, who provided information on their birthplaces. For 7543 participants, the birth location could not be accurately mapped by the UK Biobank. We also removed 7102 adopted individuals due to potential inaccuracies in their birth locations or birth dates. Consequently, our final sample size for the analysis is 437,504.

### Temperature data

**Fig. 2:**
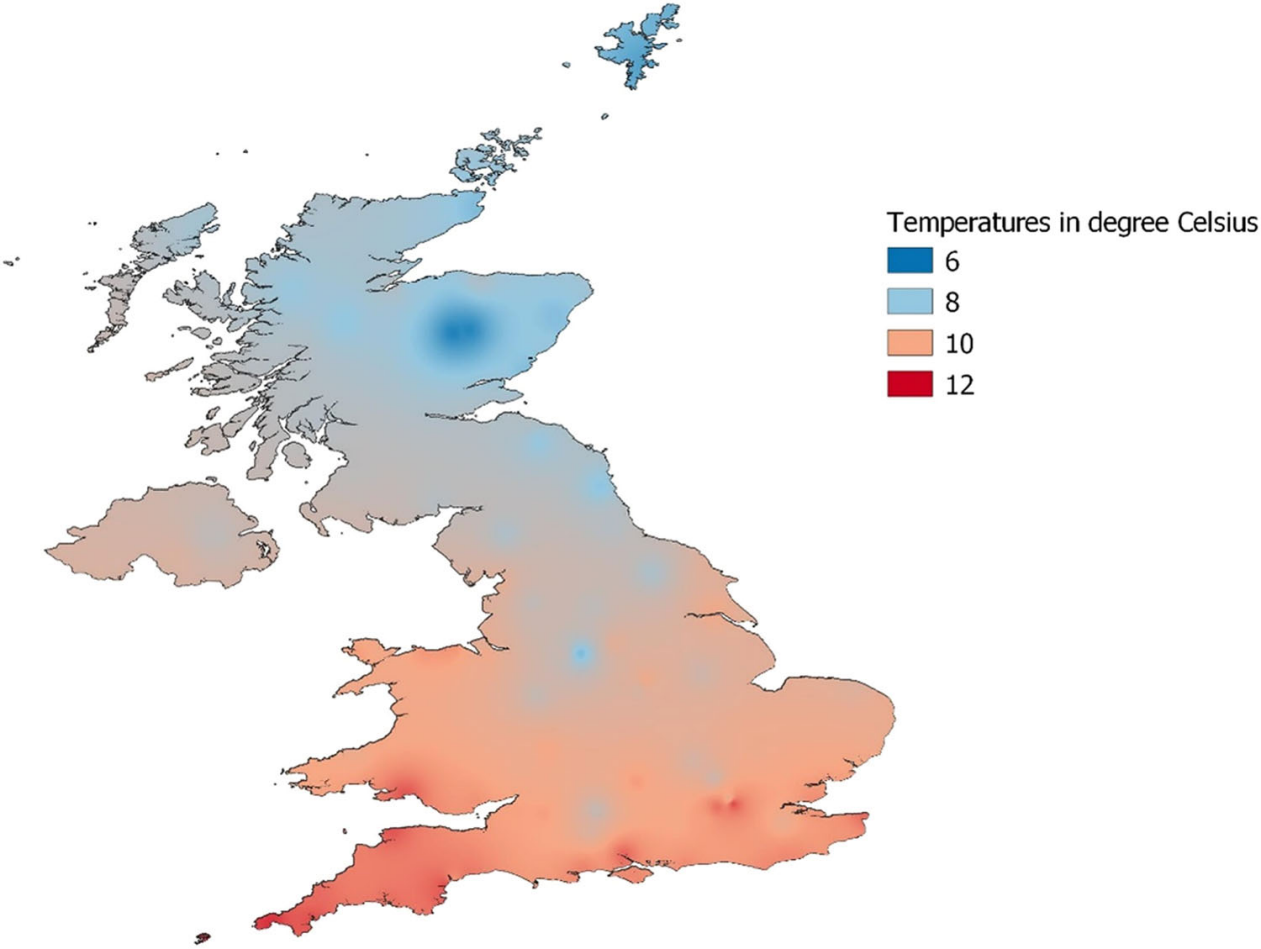
Heat map of long-run annual temperature patterns in the UK. Average annual temperatures in the UK from 1933 to 1971, based on data from 94 MIDAS Open weather stations, each with at least 30 years of recording. The average temperatures are represented using inverse distance weighting interpolation. The map is created with QGIS version 3.16.14-Hannover, using shapefiles from GADM version 3.6 (https://gadm.org/index.html).

We use temperature data from MIDAS Open UK Daily Temperature (v202107), provided by the UK’s national weather service^50^. This dataset comprises weather station-based records of daily minimum and maximum air temperature in degrees Celsius. Daily temperatures are calculated by computing the average of the daily minimum and maximum temperatures per weather station. We consider temperature records from 94 MIDAS weather stations that reported temperatures for at least 30 years between 1933 and 1971 (Fig. 1). The year 1933 is selected as the starting point, as it corresponds to the earliest expected conception year for the oldest individual in our study. Average annual temperatures across the UK spanning the period from 1933 to 1971 are presented in a heat map (Fig. 2). This study period precedes the onset of marked global temperature increases that began in the early 1980s and therefore represents a relatively stable climatic phase in the UK without major long-term warming trends (Supplementary Fig. 1).

Temperature records from weather stations within a 200-km radius of participants’ birthplaces are aggregated using an inverse-distance weighting approach. This method assigns weights based on proximity to ensure that weather stations farther away have less influence on the computation of average temperatures. In a robustness check, we employ a nearest-neighbor approach, selecting temperature records from the station closest to the participants’ birthplace. Distances are calculated using Vincenty equations^58^. In our study, the median distance to the nearest weather station is around 20 km.

### Temperature deviations from the long-term average

For each weather station, we determine a station-specific long-run temperature profile for each day of the year (1–365), averaging over the years 1933–1971 using a polynomial function of the fourth degree. To compute temperature deviations from the long-run average, differences between actual temperatures and expected temperatures are calculated for each station. Mean temperature deviations over a timeframe are then determined by equally weighing each day within the timeframe, assuming a uniform distribution. For example, an individual was conceived in Birmingham on January 15, 1963. The long-term average temperature for this date in Birmingham would be around 3 °C. However, on January 15, 1963, the actual temperature might have been notably colder than this trend, possibly reaching −10 °C (the winter of 1962/63 was one of the harshest winters in UK history, also called the “Big Freeze”). The temperature deviation on this day would thus have been −13 °C. Similar deviations are calculated for each day for the given time period, and the average of these is taken.

### Exposure: temperature before conception

To determine the estimated date of conception (DOC), we calculate 266 days backwards from the date of birth, with 266 days representing the average pregnancy length^56^. The temperature in the ca. 2 weeks preceding conception plays an essential role in epigenetic programming^52,53^. Thereby, two main challenges exist. First, to accurately capture this critical period, we must consider the natural variation in pregnancy duration. Second, the relevant exposure period before conception is likely days or weeks, rather than being limited to a single moment in time. Thus, average temperature deviations are calculated for five different timeframes: estimated date of conception (DOC), 2 weeks before conception to estimated DOC (−2 w; DOC), 3 weeks before to 1 week after (−3 w; +1 w), 4 weeks before to 2 weeks after (−4 w; +2 w), and 5 weeks before to 3 weeks after (−5 w; +3 w). Shorter time intervals enhance precision by reducing noise but may fail to capture the broader exposure patterns that encompass the entire biologically relevant period. Conversely, longer intervals are better at including the critical biological period, but they do introduce additional noise, weakening effect sizes by covering irrelevant time periods. Moreover, non-systematic errors in estimating temperatures during the critical window can bias results toward lower-bound estimates.

We observe larger effects on adults’ metabolic outcomes when using more extended timeframes – possibly because sustained temperature deviations over a longer period are more likely to overlap with the crucial epigenetic window. At the same time, longer intervals can mathematically dilute temperature deviations, potentially inflating effect estimates. For instance, if only the 2 weeks before conception truly matter and only those 2 weeks are 1° warmer, the average temperature deviation over an 8-week window (e.g., −5 w; +3 w) would be +0.25 °C, yet the associated outcome change stems entirely from those two warmer weeks, artificially magnifying the estimated effect size. In light of these trade-offs, we employ both narrow and broad exposure definitions to balance precision with the need to capture the entire biologically relevant period. Consequently, some of our timeframes extend beyond the 2 weeks prior to the estimated date of conception; however, since paternal (rather than maternal) cold exposure appears essential^13^, ambient temperatures after conception should have little impact on offspring BAT.

### Empirical strategy

We regress metabolic outcomes on temperatures around conception. To address potential confounding through place and season of conception, we use area- and day-of-the-year-specific temperature deviations, in combination with area-by-month of birth fixed effects in all regressions. Identifying the effect of temperature from the randomness in temperature variation (as opposed to absolute temperatures) allows us to provide more robust evidence on the link between pre-conception temperature and later-life metabolic health outcomes.

We employ the following linear regression model to examine this relationship:

1 $${Metaboli}{c}_{{irt}}=\alpha {TempDe}{v}_{{irt}}+{\gamma }_{{rm}}+{{{\boldsymbol{X}}}}_{{irt}}{\prime} \beta +{\varepsilon }_{{irt}}$$

In this model, *Metabolic* represents metabolic health outcomes (BMI, waist circumference, HbA1c, triglycerides, and total cholesterol levels) of individuals (indexed by *i*) conceived in area *r* at time point (date) *t*. The coefficient *α* is central to our analysis, representing the effect of *TempDev*, temperature deviation from the long-term, area-specific trend before conception. A negative *TempDev* signifies that temperatures were colder than usual for the individual’s birthplace’s location and day of year during the respective timeframe before conception. Area-by-month of birth fixed effects γ_rm_ capture time-invariant area-level and seasonal factors influencing metabolic outcomes. A set of socio-demographic characteristics, including sex, year of birth, and year of assessment, is represented in vector ***X***_*irt*_. Controlling for the year of birth and year of assessment helps account for general time trends in metabolic outcomes. However, no later-life characteristics, such as the socio-economic status, were controlled for, since these factors could themselves be influenced by the exposure of interest. Including them could introduce bias, known as the “bad control” problem. The stochastic error term is represented by ${\varepsilon }_{{irt}}$, with standard errors clustered at the area-level to account for correlations within areas. Areas are defined as larger administrative areas, including counties and districts/boroughs within the UK. In total, the UK is divided into 172 administrative areas.

We also analyzed whether temperature deviations prior to conception are linked with a heightened risk of metabolic conditions in adulthood. To do so, we generate binary indicators of metabolic health using established cut-off points by the International Diabetes Federation^59^ and the American Diabetes Association^60^, as described in the results section, and employ these as outcome variables within a logistic regression framework. The regression model is as follows, where Λ represents the cumulative distribution function of the standard logistic distribution:

2 $$	\Pr \left({HigherRisk} = 1\,\Big|{TempDev},\gamma ,{{\boldsymbol{X}}}\right) \\ 	 = \Lambda \left(\alpha {TempDe}{v}_{{irt}}+{\gamma }_{{rm}}+{{{\boldsymbol{X}}}}_{{irt}}^{\prime} \beta +{\varepsilon }_{{irt}}\right)$$

### Statistics and reproducibility

Statistical analyses are conducted using linear and logistic regression models, as described above. For continuous metabolic outcomes, we estimate linear regressions with area-clustered standard errors. For binary indicators of metabolic health risk, we employ logistic regression models. All models include area-by-month of birth fixed effects and control variables for sex, year of birth, and year of assessment. The final sample consists of 437,504 participants from the UK Biobank. Each participant represents one observation, and no repeated measures or laboratory experiments are involved. All analyses are performed using Stata version 18, with two-sided *p*-values <0.05 considered statistically significant.

### Ethics approval

This research was conducted using data from the UK Biobank resource under Application Number 74910. The UK Biobank study was approved by the North West Multi-centre Research Ethics Committee (Ref11:/NW/0382). All participants provided written informed consent.

## Results

### Sample characteristics

Relevant summary statistics for our final sample are presented in Table 1. The participants have a mean age of 56.7 years, with 54.1% being female. According to cut-off points indicating a heightened risk of developing cardiovascular disease or type 2 diabetes—as established by the International Diabetes Federation^59^ and the American Diabetes Association^60^—our final sample exhibits the following characteristics: 67.1% are categorized as overweight, defined as having a BMI of 25 kg/m² or higher, and 60.7% have a large waist circumference (≥94 cm for men and ≥80 cm for women). Pre-diabetes or diabetes, indicated by HbA1c levels of 39 mmol/mol or higher, is present in 17.4% of participants. Elevated triglyceride levels (≥1.7 mmol/l) are found in 40.5% of the sample, and 66.4% have high total cholesterol levels (≥5.2 mmol/l).

**Table 1:** Metabolic outcomes and demographic characteristics of UK Biobank participants

	Mean	Std. dev.	Observations
**Metabolic outcomes (continuous)**			
BMI (kg/m²)	27.442	4.789	435,873
Waist circumference (cm)	90.339	13.512	436,542
HbA1c (mmol/mol)	35.969	6.519	408,742
Triglycerides (mmol/mol)	1.755	1.025	410,103
Total cholesterol (mmol/l)	5.711	1.144	410,431
**Metabolic outcomes (binary)**			
Overweight	0.671	0.470	435,873
High waist circumference	0.607	0.488	436,542
High HbA1c	0.174	0.379	408,742
High triglycerides	0.405	0.491	410,103
High cholesterol	0.664	0.472	410,431
**Demographic characteristics**			
Age (years)	56.710	8.034	437,504
Female sex (versus male)	0.541	0.498	437,504

Descriptive statistics for UK Biobank participants included in the analyses (*n* = 437,504). The binary metabolic outcome variables refer to classifications for being overweight (BMI ≥25 kg/m²), having elevated waist circumference (≥94 cm for men and ≥80 cm for women), high HbA1c levels (≥39 mmol/mol), elevated triglycerides levels (≥1.7 mmol/l), and high cholesterol levels (≥5.2 mmol/l) according to the International Diabetes Federation and the American Diabetes Association^59,60^.

### Main associations between pre-conception temperature and metabolic health

**Fig. 3:**
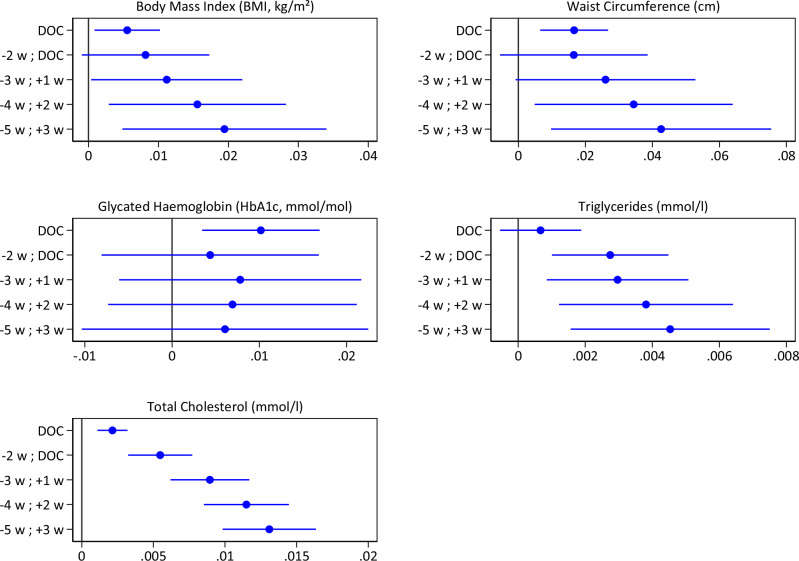
Impact of a 1 °C increase in ambient temperature around conception on adult metabolic outcomes. This figure illustrates the effects of a 1 °C increase in ambient temperature in the indicated time windows around conception (w = week) on metabolic outcomes in adulthood, presented as point estimates with 95% confidence intervals. The estimated date of conception (DOC) is calculated as the birth date minus 266 days. Temperature deviations are assigned to UK Biobank participants using inverse distance weighting from weather stations within a 200-km radius of birthplaces. Model covariates: area-by-month of birth fixed effects, sex, year of birth, and year of assessment. Standard errors are clustered at the area level (172 areas).

We find that negative pre-conceptional temperature deviations—that is, when individuals were conceived during colder-than-usual periods for a specific time and place—lead to improved metabolic outcomes in adulthood: reduced BMI, waist circumference, triglyceride levels, and total cholesterol levels (Fig. 3). For each of these four outcomes, effect sizes are larger when we choose a longer periconceptual exposure period. For the longest time period, 5 weeks before till 3 weeks after DOC, a 1 °C temperature increase translates into BMI increases of 0.019 kg/m^2^ (0.41% of a standard deviation), waist circumference increases of 0.043 cm (0.32% of a standard deviation), total cholesterol increases of 0.013 mmol/l (1.14% of a standard deviation), and increases in triglycerides of 0.005 mmol/l (0.44% of a standard deviation). For HbA1c, there was only evidence for an effect for temperature at DOC, where we observed an increase in 0.010 mmol/mol corresponding to 0.16% of a standard deviation.

Because of testing multiple outcomes, we additionally report corrected *p*-values for each time period using the Bonferroni-Holm method (Table 2). This does not change the overall conclusions. Analyses using the nearest-neighbor approach instead of inverse distance weighting yield very similar results (Supplementary Fig. 2). Therefore, our findings suggest that colder-than-usual temperatures around conception are linked to improved metabolic outcomes in later life.

**Table 2:** Impact of a 1 °C increase in ambient temperature around conception on adult metabolic outcomes for different time windows

	(1)	(2)	(3)	(4)	(5)
	Metabolic outcomes				
Time window before conception	BMI	WC	HbA1c	Triglycerides	Total Cholesterol
Date of conception (DOC)	0.006	0.017	0.010	0.001	0.002
	(0.002)	(0.005)	(0.003)	(0.001)	(0.001)
Corrected *p*	0.039	0.005	0.008	0.276	<0.001
2 weeks before conception till DOC	0.008	0.017	0.004	0.003	0.005
	(0.005)	(0.011)	(0.006)	(0.001)	(0.001)
Corrected *p*	0.232	0.277	0.488	0.007	<0.001
3 weeks before till 1 week after DOC	0.011	0.026	0.008	0.003	0.009
	(0.005)	(0.014)	(0.007)	(0.001)	(0.001)
Corrected *p*	0.122	0.111	0.266	0.022	<0.001
4 weeks before till 2 weeks after DOC	0.016	0.034	0.007	0.004	0.011
	(0.006)	(0.015)	(0.007)	(0.001)	(0.002)
Corrected *p*	0.046	0.043	0.337	0.015	<0.001
5 weeks before till 3 weeks after DOC	0.019	0.043	0.006	0.005	0.013
	(0.007)	(0.017)	(0.008)	(0.002)	(0.002)
Corrected *p*	0.026	0.021	0.466	0.010	<0.001
Mean	27.442	90.339	35.969	1.755	5.711
SD	4.789	13.512	6.519	1.025	1.144
*N*	435,873	436,542	408,742	410,103	410,431

The five timeframes, ranging from the estimated date of conception (DOC) to up to 5 weeks before till 3 weeks after DOC, are designed to capture the effects of temperature exposure during both immediate and extended pre-conception periods on later outcomes. Temperature deviations are assigned to UK Biobank participants using inverse distance weighting from stations within a 200-km radius of birthplaces. *p*-values of regression coefficients are from two-sided *t*-tests within linear regression models. Model covariates: area-by-month of birth fixed effects, sex, year of birth, and year of assessment. Standard errors clustered at the area-level (172 areas) are given in parentheses. The table also presents the mean and standard deviation (SD) of each outcome. *p*-values have been corrected for multiple testing for each time period using the Bonferroni-Holm method. Exact *p*-values (rounded to three decimals) are provided where possible; for very small values, *p* < 0.001 is reported.

*BMI* Body Mass Index, *WC* Waist Circumference, *HbA1c* Glycated Hemoglobin.

### Robustness, sensitivity, and additional analyses

When using binary outcome variables, negative temperature deviations are associated with lower odds of adverse metabolic health outcomes in adulthood (Supplementary Fig. 3). We employ this approach since it allows for a better understanding of whether exposure to colder-than-usual temperatures before conception decreases the likelihood of reaching elevated cardiovascular risk levels. Moreover, it serves as a robustness check to capture potential non-linearities in the effects of temperature deviations. Notably, the results show a high degree of similarity to those obtained using the continuous versions of metabolic health indicators. We also conduct separate analyses for women (Supplementary Fig. 4) and men (Supplementary Fig. 5). While the overall patterns remain consistent between both sexes, the effect sizes appear stronger for waist circumference in men, although sex-specific estimates have lower precision. Furthermore, a separate analysis for winter conceptions (individuals conceived between October and March) is performed to investigate whether colder temperatures matter more when absolute temperatures are relatively low. The findings, presented in Supplementary Fig. 6, indicate that the effects do not differ substantially from our main results (Fig. 3).

A potential concern is that deviations from typical temperatures for a given location and time of the year could be linked to the background characteristics of those conceiving a child. Through robustness checks, we assess whether selective fertility could have substantially biased our results. First, we exclude participants born to mothers younger than 20 years old, as their fertility patterns are possibly more responsive to ambient temperature patterns. Secondly, we control for whether the mother smoked around the time of birth, as this is correlated with socioeconomic status. Even though information on maternal smoking is only available for a smaller sample and, consequently, coefficients are estimated with larger standard errors, effect patterns remain similar. A change in our coefficients of interest when controlling for maternal smoking would suggest a correlation between temperature variations and the types of people who are conceiving. In both analyses, the results remain robust compared to those from our main analyses (Supplementary Fig. 7 and Supplementary Fig. 8). As a negative control test, we further examine whether temperature before conception is associated with two outcomes that should not plausibly be affected by temperature: self-reported birth weight and the total number of siblings from the same parents. The idea behind this test is that any association between temperature exposure and these outcomes would almost certainly reflect spurious correlation rather than a causal effect. As shown in Supplementary Fig. 9, the estimated effects are very close to zero, and there is no evidence for an effect, which provides further reassurance that our main findings are unlikely to be driven by residual confounding.

Results from analyses that include month-by-year of birth, as well as area fixed effects, instead of area-by-month of birth fixed effects, further support our results. These analyses take out potentially residual confounding due to time effects. However, these results are considerably less precise due to a loss of most of the variation of interest in temperature deviations (Supplementary Fig. 10).

Additional analyses (Supplementary Fig. 11) indicate that the associations between pre-conception temperature deviations and adult metabolic outcomes seem to be stronger for conceptions occurring between October and December than for those between January and March. This suggests that the timing of conception within the cold season may influence the magnitude of these effects, potentially reflecting differences in parental physiological adaptation to cold conditions.

Results remained robust across several additional checks. Excluding participants of non-White ethnicity, who may be more likely to have been conceived outside the UK (*n* = 7594), did not affect the results. Likewise, using finer regional definitions based on 3^rd^-level administrative units (“wards” or “electoral divisions”) produced virtually identical results. These analyses are not reported, as the findings were unchanged.

## Discussion

Utilizing a comprehensive dataset of measurements and biomarkers from over 437,500 participants, we show that lower ambient temperatures around conception lead to better metabolic outcomes in adult humans. We use a natural experiment approach that aims to get close to causality in a setting where randomized controlled trials are not feasible. By leveraging quasi-random temperature deviations from location-specific average temperatures before conception and controlling for area and month of birth, we minimize potential confounding due to selective fertility and residential patterns. We conduct robustness checks, which indicate that these findings are unlikely to be driven by selective fertility. This paper thus provides empirical evidence for fetal programming in response to cold exposure around conception, supporting the hypothesis that pre-conception temperatures can have lasting impacts on health.

Our findings indicate that colder-than-usual temperatures around conception are associated with improved metabolic outcomes and a lower metabolic risk later in life. Sun et al.^13^ report that humans conceived during the colder months of the year are more likely to have active BAT in adulthood. With our study, we are the first to establish a causal link between temperature exposure around conception and adult metabolic health outcomes. We do so using a broad range of metrics – BMI, waist circumference, HbA1c, and blood lipid levels (triglycerides and cholesterol). Although we are unable to distinguish between maternal and paternal temperature exposure, previous research emphasizes the critical role of paternal exposure, particularly highlighting the 2 weeks before conception as a crucial period for epigenetic programming in sperm cells^13,52,53^.

Our research faces certain challenges and limitations. We do not know the exact date of conception, and it remains unclear which specific period around conception is most critical for epigenetic programming. To address this, we calculate temperature deviations for five different time windows, using both narrow and broad intervals. Narrow intervals provide higher precision but risk missing part of the biologically relevant window, while broader intervals capture a longer timeframe but may include irrelevant exposures. Any non-systematic errors in estimating temperature deviations—due to uncertainty in conception date and location—tend to dilute effect sizes (attenuation bias). On the other hand, choosing broader time windows can also mathematically inflate effect estimates by averaging smaller deviations over longer periods. Further discussion on the direction of a potential bias can be found in the “Methods” section.

Moreover, we do not know the exact location of conception and therefore use the place of birth as a proxy. Place of birth may deviate from place of conception, and place of birth itself may be reported with error^61^. This leads to non-differential measurement error and thus bias toward the null value.

The average age of respondents was 57 years, potentially leading to survival bias. If those with the worst metabolic health parameters were more likely to have died before recruitment or declined participation, this would additionally attenuate the estimated associations toward the null value.

Further, ambient temperature does not directly translate to the temperature actually experienced by individuals. People spend varying amounts of time indoors, where homes are often heated, and wear different types of clothing, leading to variability in experienced temperatures. One can assume that if people were fully exposed to the weather, the effects found here would have been larger.

It is important to recognize that both indoor and outdoor temperatures in the UK have been steadily increasing. Outdoor temperature rises are primarily driven by climate change, while indoor temperature increases result from advancements in home insulation and heating practices over the years. Additionally, people tend to spend more time indoors nowadays than a few decades ago^62^. These trends may have significant implications for future metabolic health outcomes, as elevated indoor temperatures may reduce the beneficial exposure to colder temperatures that our study highlights.

To provide a sense of magnitude, we provide a set of illustrative “back-of-the-envelope” calculations to gauge how changes in temperatures around conception could affect metabolic outcomes several decades later. These calculations are admittedly imprecise based on the points raised above and should only serve as a frame of reference. Furthermore, these estimates assume that all factors related to temperature exposure remain as defined in this study. For example, the amount of time individuals spend outdoors and thus exposed to ambient temperatures does not change over time. Since our regression estimates the effect size for each 1 °C increase in temperature, a climate change scenario with a 2°C rise can be modeled by doubling the regression coefficients. A 2 °C increase in ambient temperatures would lead to an increase in body weight of 126 g (95% confidence interval (CI): 31–220 g) for an individual of 1.80 m height and a body weight of 80 kg. Total cholesterol levels for such a person would increase by 2.29% of a standard deviation (95% CI: 1.72%–2.86%).

Increases in average indoor temperatures in the UK amounted to 6.5 °C since 1970^63^. Partially this is due to the adoption of central heating (present in 5% of UK homes in 1960 and 90% in 2011), and partially due to better insulation (cavity wall insulation being present among 2% of UK households in 1974 and 64% in 2011)^63,64^. Assuming the same temperature–outcome relationship as above, the effects would be more than three times as large: an increase in body weight by 409 g (95% CI: 102–717 g) and an increase in total cholesterol by 7.44% of a standard deviation (95% CI: 5.59%–9.29%).

Besides shedding light on the impact of pre-conception temperature exposure on long-term metabolic health, our study also points to some directions for future research. Measurements of BAT activity are not included in the UK Biobank and are usually not available in large biodata repositories. As a result, we could only observe metabolic outcomes consistent with higher BAT activity, but could not measure BAT activity itself. It would be helpful if BAT activity could be added to data collection in the future.

Second, our study built on findings from previous research suggesting that the final 1–2 weeks of spermatogenesis are the most sensitive period for temperature exposure. Future research should establish the exact time frame that matters and how long an exposure to cold temperatures is needed to exert an effect.

Finally, the potential role of epigenetic mechanisms in shaping seasonal patterns of metabolic outcomes deserves further consideration. When parents acclimatize to cold conditions, this may lead to epigenetic changes in genes involved in thermogenic pathways, which could in turn affect offspring metabolism and BAT activity^65,66^. Additional analyses suggest that the associations between temperature deviations before conception and later-life metabolic outcomes seem stronger for conceptions occurring between October and December than for those between January and March. This pattern may reflect that parents are less adapted to cold early in winter, making unexpected temperature drops more physiologically relevant. As the season progresses, continued exposure to cold may result in acclimatization and possibly smaller physiological impacts of colder-than-usual spells. This interpretation is consistent with evidence showing seasonal changes in BAT activity and cold-induced thermogenesis in humans^67,68^. This highlights an important avenue for future research examining how parental seasonal adaptation and epigenetic changes influence offspring metabolic outcomes.

In summary, our research highlights that even relatively subtle prenatal environmental factors, like day of the year and place-specific variations in temperature before conception, can have substantial implications for adult metabolic health. These findings thus underline the long-term health impacts of prenatal conditions.

## Supplementary information

Supplementary Information

## Data Availability

This study utilizes data from the UK Biobank, which is subject to access restrictions. Per UK Biobank policies, individual-level data cannot be publicly shared. Individual-level data from UK Biobank are available upon application to the UK Biobank. We use temperature data from MIDAS Open: UK Daily Temperature Data (v202107), provided by UK’s national weather service (Met Office) and accessed via the Centre for Environmental Data Analysis (CEDA) archive (https://archive.ceda.ac.uk/). These data are publicly available, subject to registration with CEDA. The numerical source data underlying Fig. 3, which presents the main results of this study, are provided as Supplementary Data in Excel format.

## Code availability

The code used to generate the results in this study is publicly available at GitHub^69^.
