# Supplementary material for: Temperatures around conception affect metabolic health in adulthood
Source: Commun Med (Lond). 2026 Mar 27;6:172. doi: 10.1038/s43856-026-01496-8 (PMC13031311; doi:10.1038/s43856-026-01496-8)
Supplement: Supplementary file 1 — Supplementary Information [file 43856_2026_1496_MOESM1_ESM.pdf]

Supplementary Information (SI) for

Temperatures around conception affect metabolic health in adulthood

Timo S. Münz[1], Fabienne Pradella[1,2,3], Nathalie J. Lambrecht[4,5,6], Sabine Gabrysch[2,4,5], Reyn van Ewijk[1]*

1 Chair of Statistics and Econometrics, Johannes Gutenberg University Mainz, 55122 Mainz, Germany

2 Heidelberg Institute of Global Health, Heidelberg University, 69120 Heidelberg, Germany

3 Division of Primary Care and Population Health, Department of Medicine, Stanford University, 94305 Stanford, CA, USA

4 Charité—Universitätsmedizin Berlin, Corporate Member of Freie Universität Berlin and Humboldt-Universität zu Berlin, Institute of Public Health, 10117 Berlin, Germany

5 Research Department 2, Potsdam Institute for Climate Impact Research (PIK), Member of the Leibniz Association, 14473 Potsdam, Germany

6 Center for Innovation in Global Health, Stanford University, 94305 Stanford, CA, USA

*Corresponding Author

Name: Reyn van Ewijk

Address: Jakob-Welder-Weg 4, 55128 Mainz, Germany

Phone number: +49 (0) 6131 / 39 - 24790

Email: vanewijk@uni-mainz.de

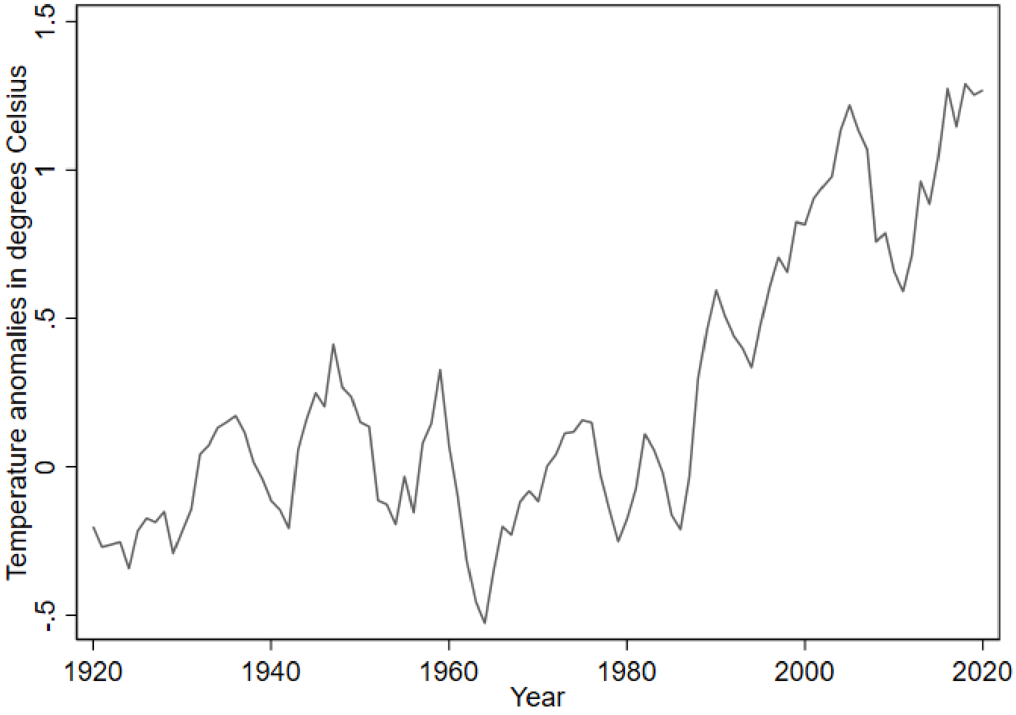

Supplementary Figure 1. Temperature Anomalies in the UK. This chart shows the five-year moving average of annual temperature deviations in the United Kingdom, measured in degrees Celsius, relative to the 1933-1971 historical regional baseline. The data are derived from the 94 MIDAS Open weather stations, each with at least 30 years of recorded observations during this period, used for the analyses.

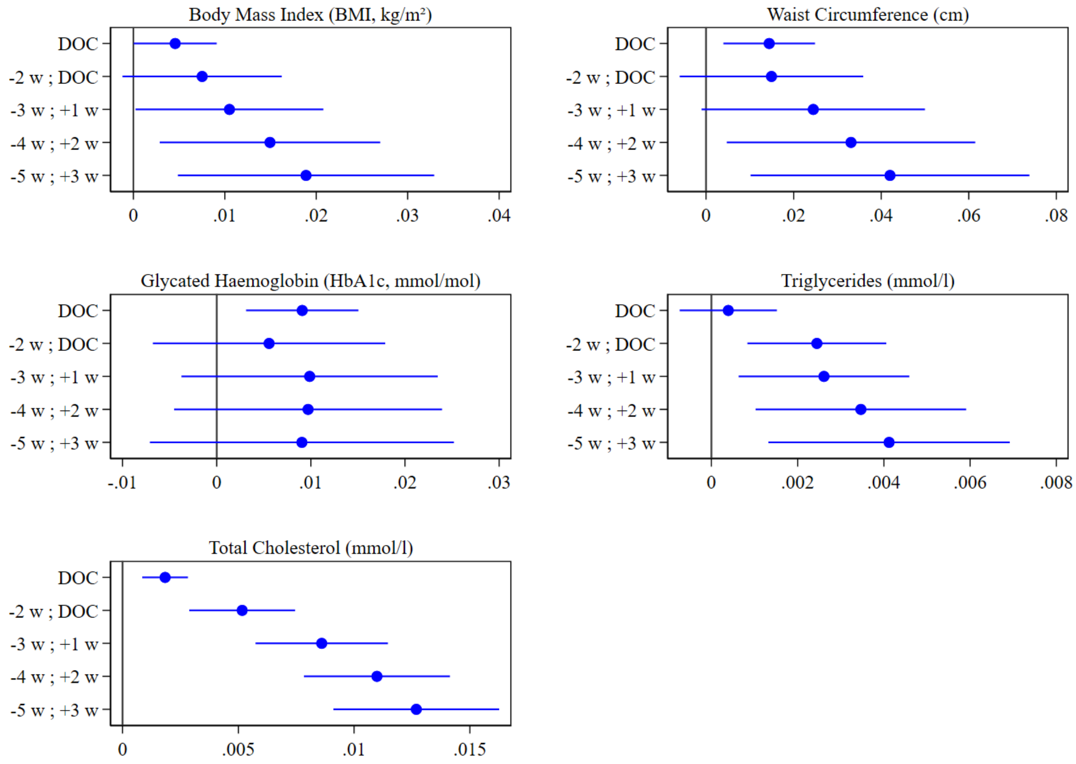

Supplementary Figure 2. Impact of a 1-degree Celsius increase in ambient temperature around conception on late adulthood metabolic outcomes using a nearest-neighbor approach. This figure illustrates the effects of a 1°C increase in ambient temperature in the indicated time windows around conception (w = week) on metabolic outcomes in adulthood, presented as point estimates with 95% confidence intervals. The estimated date of conception (DOC) is calculated as the birth date minus 266 days. Temperature deviations are linked to each participant using the nearest weather station, with a median distance of 16.4 km. Model covariates: area-by-month of birth fixed effects, sex, year of birth and year of assessment. Standard errors are clustered at the area-level (172 areas).

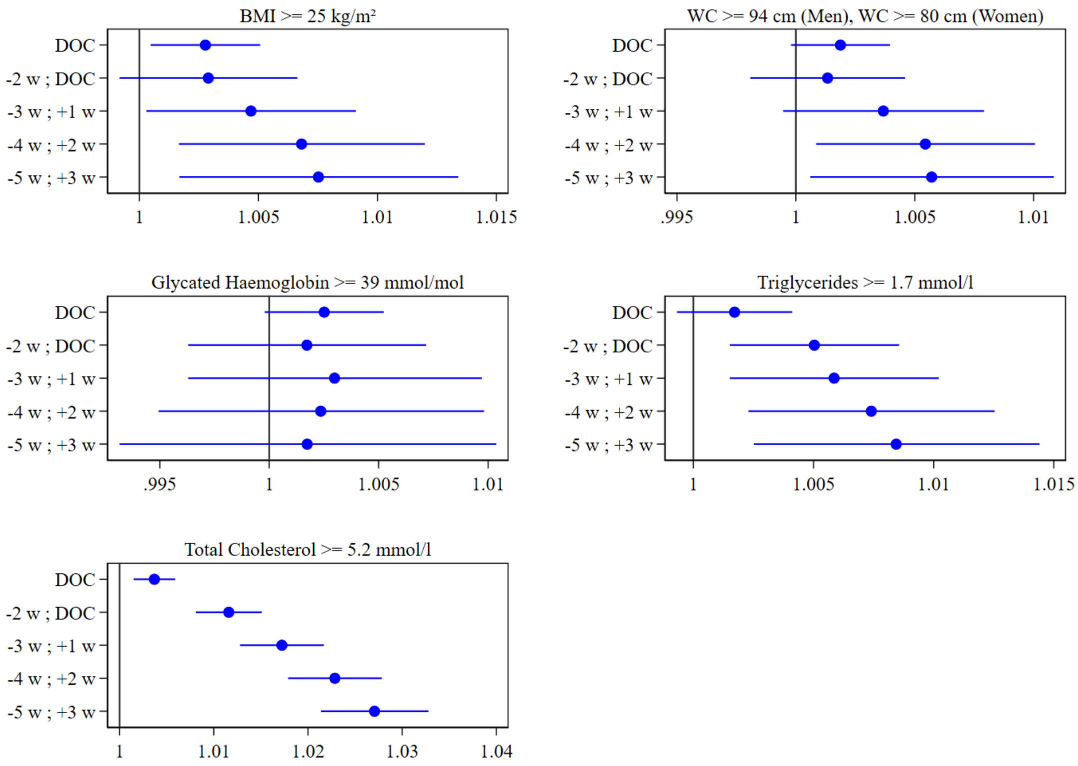

Supplementary Figure 3. Impact of a 1-degree Celsius increase in ambient temperature around conception on binary outcomes for metabolic risk in adulthood. This figure displays odds ratios from a logistic regression for a 1°C increase in ambient temperature in the indicated time windows around conception (w = week), presented as point estimates with 95% confidence intervals. The estimated date of conception (DOC) is calculated as the birth date minus 266 days. The binary outcomes assess metabolic risk, with cut-off values according to the International Diabetes Federation and American Diabetes Association (see results section). Temperature deviations before conception are assigned to each UK Biobank participant using inverse distance weighting from stations within a 200-km radius of birthplaces. Model covariates: area-by-month of birth fixed effects, sex, year of birth and year of assessment. Standard errors are clustered at the area-level (172 areas). Abbreviations: BMI (Body Mass Index), WC (Waist Circumference), HbA1c (Glycated Haemoglobin).

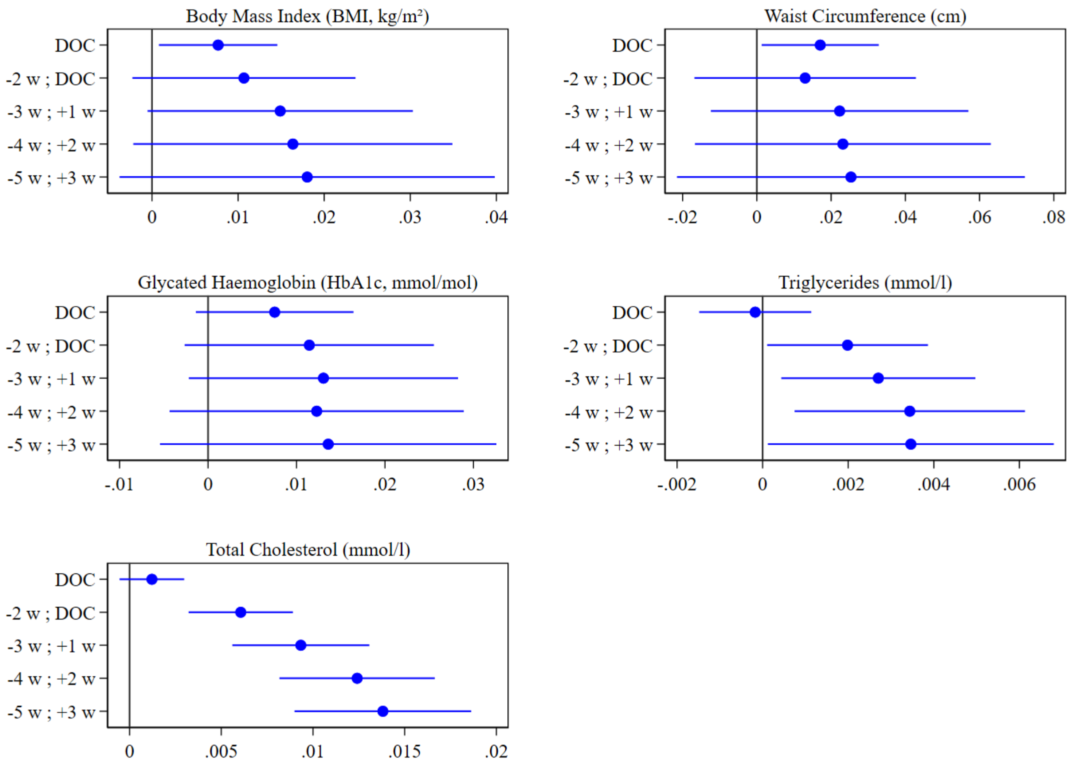

Supplementary Figure 4. Impact of a 1-degree Celsius increase in ambient temperature around conception on adult metabolic outcomes in women. This figure illustrates the effects of a 1°C increase in ambient temperature in the indicated time windows around conception (w = week) on metabolic outcomes in adulthood, presented as point estimates with 95% confidence intervals. The estimated date of conception (DOC) is calculated as the birth date minus 266 days. Temperature deviations are assigned to UK Biobank participants using inverse distance weighting from stations within a 200-km radius of birthplaces. Sample is restricted to women. Model covariates: area-by-month of birth fixed effects, year of birth and year of assessment. Standard errors are clustered at the area-level (172 areas).

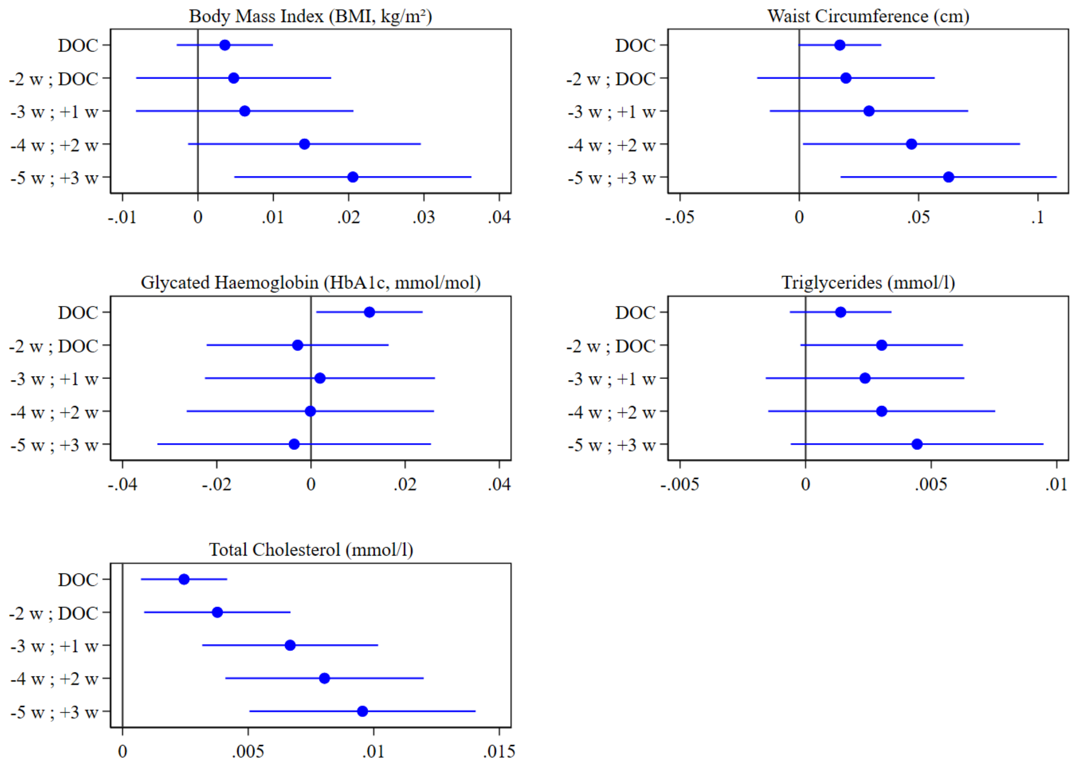

Supplementary Figure 5. Impact of a 1-degree Celsius increase in ambient temperature around conception on adult metabolic outcomes in men. This figure illustrates the effects of a 1°C increase in ambient temperature in the indicated time windows around conception (w = week) on metabolic outcomes in adulthood, presented as point estimates with 95% confidence intervals. The estimated date of conception (DOC) is calculated as the birth date minus 266 days. Temperature deviations are assigned to UK Biobank participants using inverse distance weighting from stations within a 200-km radius of birthplaces. Sample is restricted to men. Model covariates: area-by-month of birth fixed effects, year of birth and year of assessment. Standard errors are clustered at the area-level (172 areas).

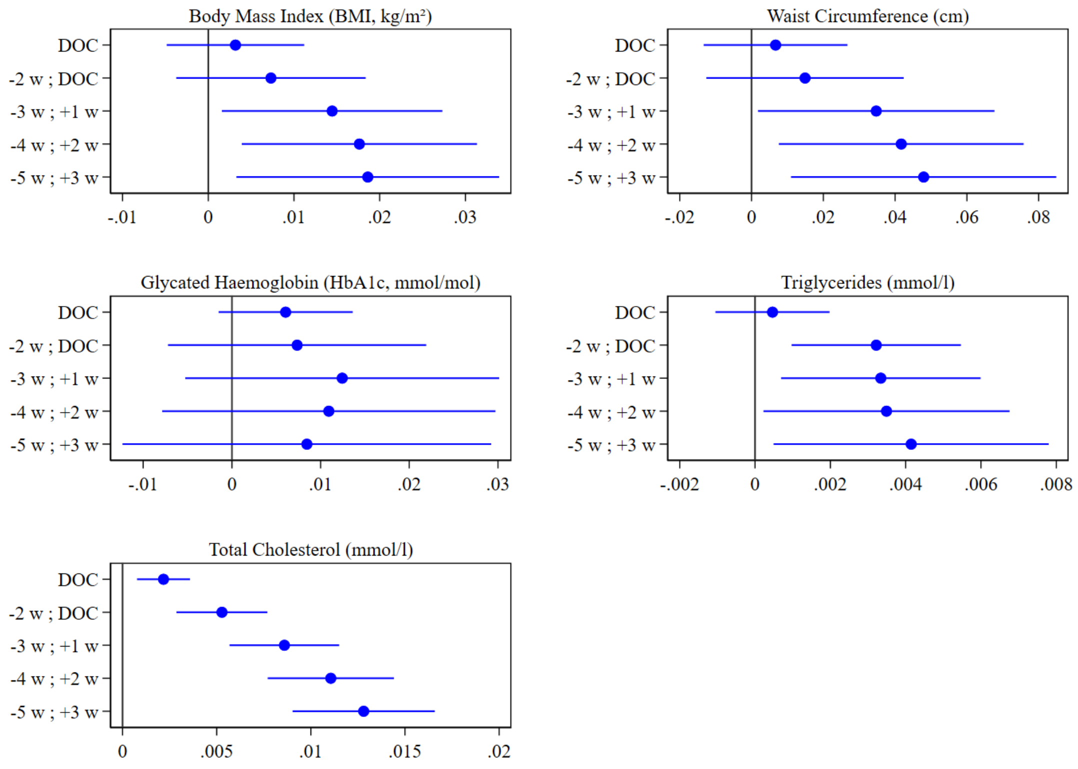

Supplementary Figure 6. Impact of a 1-degree Celsius increase in ambient temperature around conception on adult metabolic outcomes – restricted to winter conceptions (October to March). This figure illustrates the effects of a 1°C increase in ambient temperature in the indicated time windows around conception (w = week) on metabolic outcomes in adulthood, presented as point estimates with 95% confidence intervals. The estimated date of conception (DOC) is calculated as the birth date minus 266 days. Temperature deviations are assigned to UK Biobank participants using inverse distance weighting from stations within a 200-km radius of birthplaces. Sample is restricted to individuals who have been conceived in the winter half of the year (1st of October to 31st of March). Model covariates: area-by-month of birth fixed effects, sex, year of birth and year of assessment. Standard errors are clustered at the area-level (172 areas).

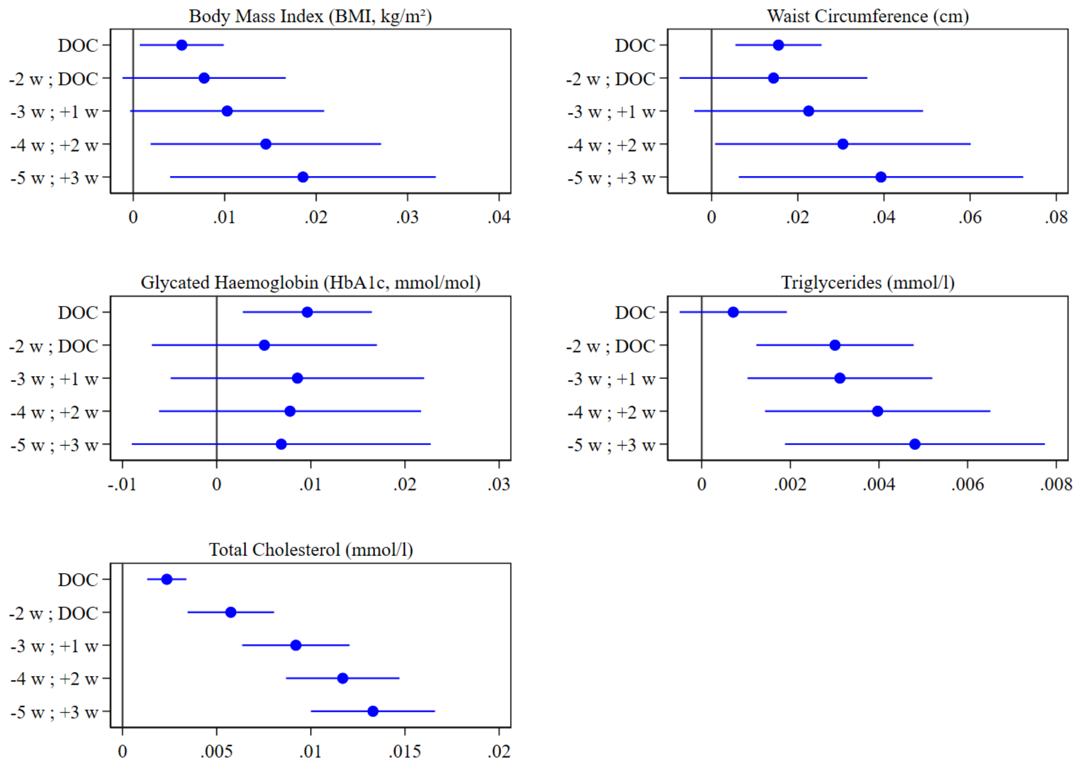

Supplementary Figure 7. Impact of a 1-degree Celsius increase in ambient temperature around conception on late adulthood metabolic outcomes – excluding teenage pregnancies. This figure illustrates the effects of a 1°C increase in ambient temperature in the indicated time windows around conception (w = week) on metabolic outcomes in adulthood, presented as point estimates with 95% confidence intervals. The estimated date of conception (DOC) is calculated as the birth date minus 266 days. Temperature deviations are assigned to each UK Biobank participant using inverse-distance weighting from stations within a 200-km radius of birthplaces. Participants with mothers younger than 20 years at birth are excluded. Model covariates: area-by-month of birth fixed effects, sex, year of birth and year of assessment. Standard errors are clustered at the area-level (172 areas).

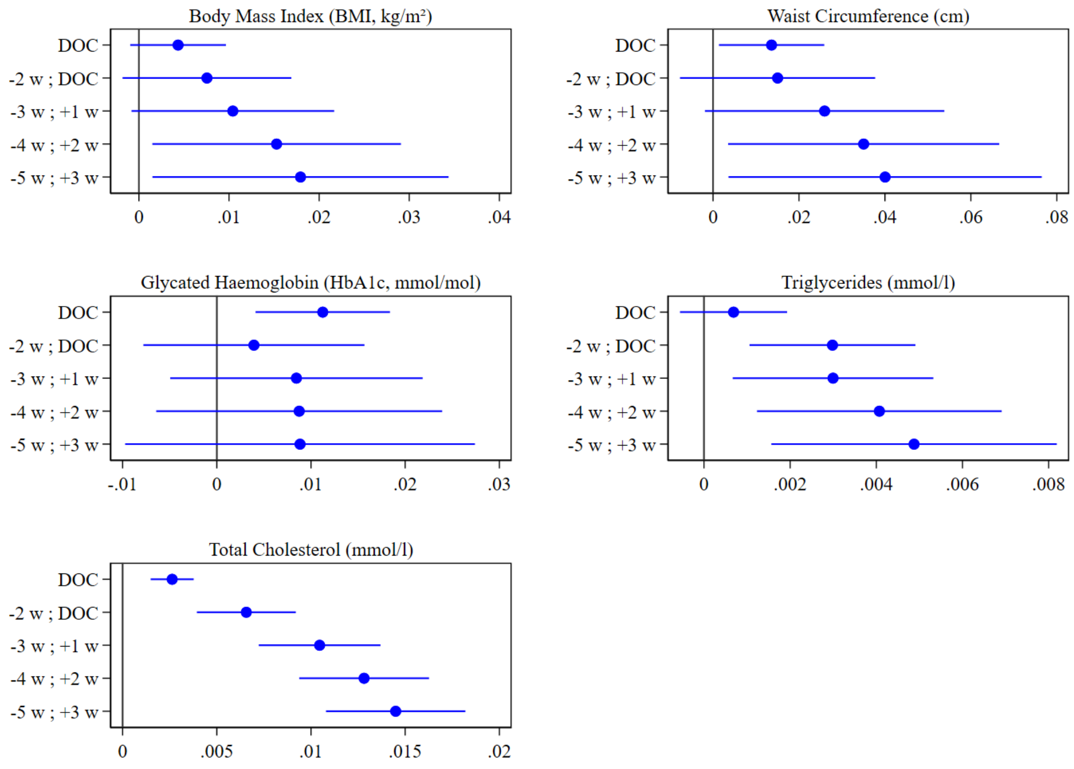

Supplementary Figure 8. Impact of a 1-degree Celsius increase in ambient temperature around conception on adult metabolic outcomes – controlling for maternal smoking status around birth. This figure illustrates the effects of a 1°C increase in ambient temperature in the indicated time windows around conception (w = week) on metabolic outcomes in adulthood, presented as point estimates with 95% confidence intervals. The estimated date of conception (DOC) is calculated as the birth date minus 266 days. Temperature deviations are assigned to each UK Biobank participant using inverse-distance weighting from stations within a 200-km radius of birthplaces. Model covariates: area-by-month of birth fixed effects, sex, year of birth and year of assessment, and maternal smoking status around the time of birth as a binary indicator. Maternal smoking status is based on the response to the question: "Did your mother smoke regularly around the time when you were born?". Standard errors are clustered at the area-level (172 areas).

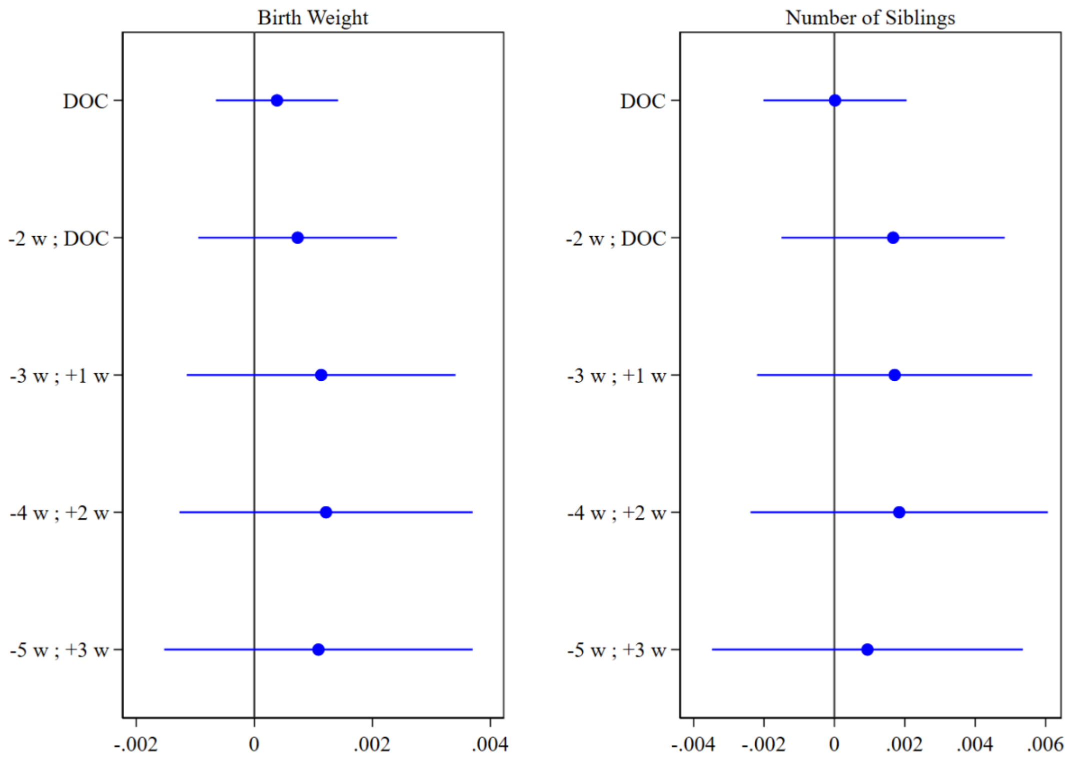

Supplementary Figure 9. Impact of a 1-degree Celsius increase in ambient temperature around conception on birth weight and number of siblings. This figure illustrates the effects of a 1°C increase in ambient temperature in the indicated time windows around conception (w = week) on birth weight and number of siblings, outcomes not plausibly affected by temperature and used as negative controls. Estimates are presented as point estimates with 95% confidence intervals. The estimated date of conception (DOC) is calculated as the birth date minus 266 days. Temperature deviations are assigned to each UK Biobank participant using inverse-distance weighting from stations within a 200-km radius of birthplaces. Model covariates: area-by-month of birth fixed effects, sex, year of birth and year of assessment. Birth weight is based on participants' self-reports, and number of siblings refers to the total number of brothers and sisters from the same parents. Standard errors are clustered at the area-level (172 areas).

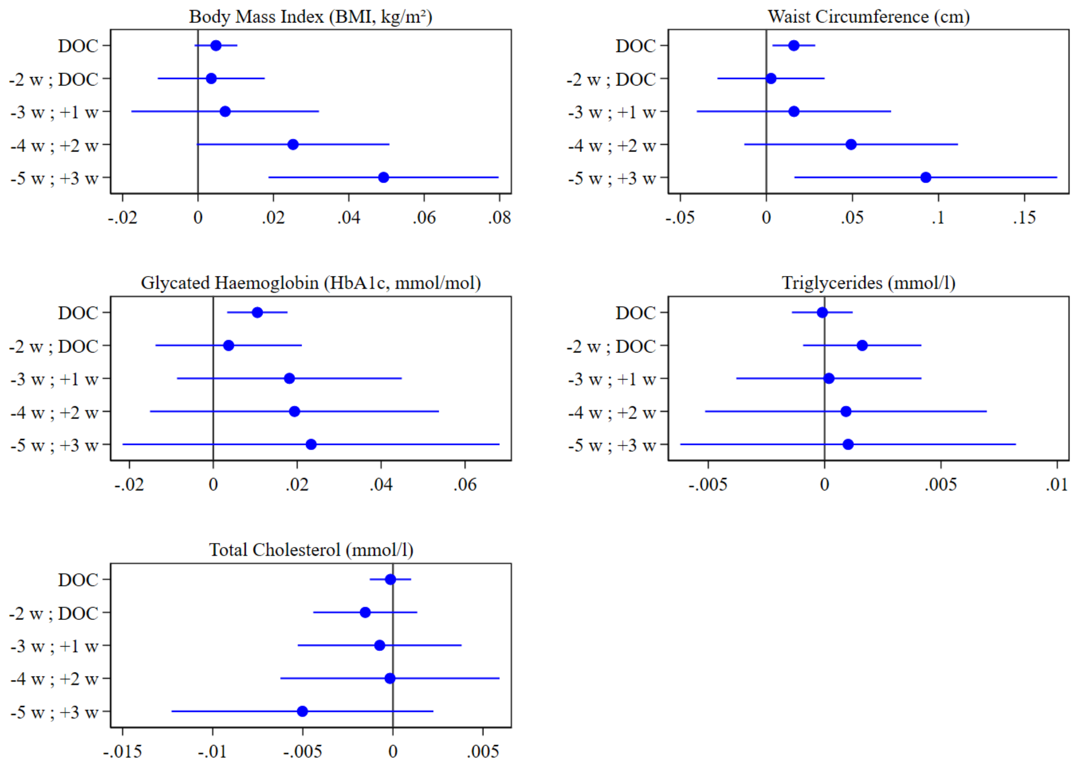

Supplementary Figure 10. Impact of a 1-degree Celsius increase in ambient temperature around conception on adult metabolic outcomes – controlling for month-by-year of birth fixed effects. This figure illustrates the effects of a 1°C increase in ambient temperature in the indicated time windows around conception (w = week) on metabolic outcomes in adulthood, presented as point estimates with 95% confidence intervals. The estimated date of conception (DOC) is calculated as the birth date minus 266 days. Temperature deviations are assigned to each UK Biobank participant using inverse-distance weighting from stations within a 200-km radius of birthplaces. Model covariates: month-by-year of birth fixed effects, area-of-birth fixed effects, sex, year of birth and year of assessment. Standard errors are clustered at the area-level (172 areas).

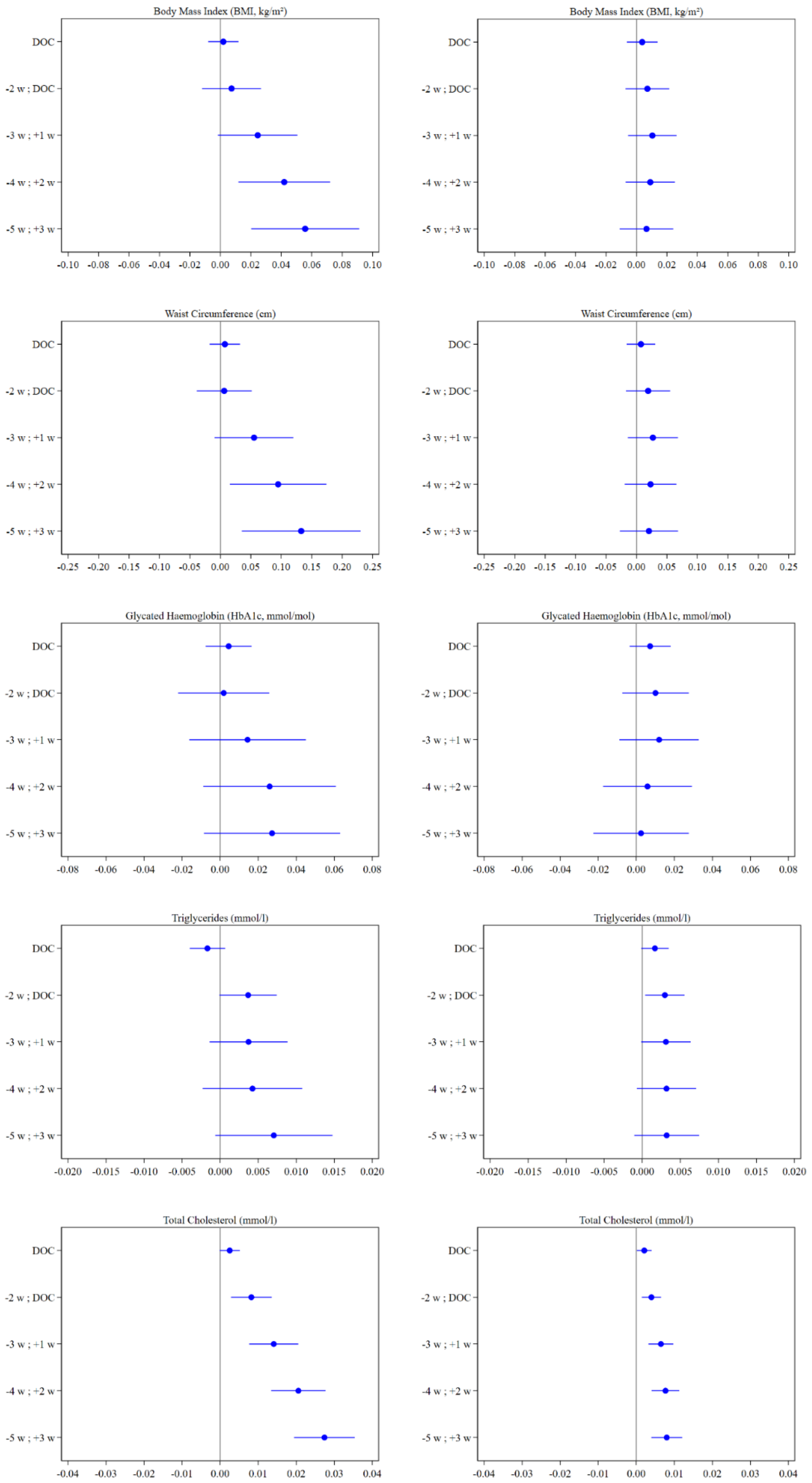
Body Mass Index (BMI, kg/m²)
DOC
-2 w ; DOC
-3 w ; +1 w
-4 w ; +2 w
-5 w ; +3 w
-0.10 -0.08 -0.06 -0.04 -0.02 0.00 0.02 0.04 0.06 0.08 0.10
Body Mass Index (BMI, kg/m²)
Waist Circumference (cm)
-0.25 -0.20 -0.15 -0.10 -0.05 0.00 0.05 0.10 0.15 0.20 0.25
Waist Circumference (cm)
Glycated Haemoglobin (HbA1c, mmol/mol)
-0.08 -0.06 -0.04 -0.02 0.00 0.02 0.04 0.06 0.08
Glycated Haemoglobin (HbA1c, mmol/mol)
Triglycerides (mmol/l)
-0.020 -0.015 -0.010 -0.005 0.000 0.005 0.010 0.015 0.020
Triglycerides (mmol/l)
Total Cholesterol (mmol/l)
-0.04 -0.03 -0.02 -0.01 0.00 0.01 0.02 0.03 0.04
Total Cholesterol (mmol/l)

Supplementary Figure 11. Impact of a 1-degree Celsius increase in ambient temperature around conception on adult metabolic outcomes, separately for October-December and January-March conceptions. This figure illustrates the effects of a 1°C increase in ambient temperature in the indicated time windows around conception (w = week) on metabolic outcomes, shown separately for two seasonal conception periods: October-December (left panel) and January-March (right panel). Estimates are presented as point estimates with 95% confidence intervals. The estimated date of conception (DOC) is calculated as the birth date minus 266 days. Temperature deviations are assigned to each UK Biobank participant using inverse-distance weighting from stations within a 200-km radius of birthplaces. Model covariates: area-by-month of birth fixed effects, sex, year of birth and year of assessment. Standard errors are clustered at the area-level (172 areas).
